# The Congruency Effect in the Posterior Medial Frontal Cortex Is More Consistent with Time on Task than with Response Conflict

**DOI:** 10.1371/journal.pone.0062405

**Published:** 2013-04-29

**Authors:** Daniel H. Weissman, Joshua Carp

**Affiliations:** Department of Psychology, University of Michigan, Ann Arbor, Michigan, United States of America; Radboud University Nijmegen, The Netherlands

## Abstract

The posterior medial frontal cortex (pMFC) is thought to play a pivotal role in enabling the control of attention during periods of distraction. In line with this view, pMFC activity is ubiquitously greater in incongruent trials of response-interference (e.g., Stroop) tasks than in congruent trials. Nonetheless, the process underlying this congruency effect remains highly controversial. We therefore sought to distinguish between two competing accounts of the congruency effect. The conflict monitoring account posits the effect indexes a process that detects conflict between competing response alternatives, which is indexed by trial-specific reaction time (RT). The time on task account posits the effect indexes a process whose recruitment increases with time on task independent of response conflict (e.g., sustained attention, arousal, effort, etc.). To distinguish between these accounts, we used functional MRI to record brain activity in twenty-four healthy adults while they performed two tasks: a response-interference task and a simple RT task with only one possible response. We reasoned that demands on a process that detects response conflict should increase with RT in the response-interference task but not in the simple RT task. In contrast, demands on a process whose recruitment increases with time on task independent of response conflict should increase with RT in both tasks. Trial-by-trial analyses revealed that pMFC activity increased with RT in both tasks. Moreover, pMFC activity increased with RT in the simple RT task enough to fully account for the congruency effect in the response-interference task. These findings appear more consistent with the time on task account of the congruency effect than with the conflict monitoring account.

## Introduction

The posterior medial frontal cortex (pMFC) is thought to participate in a process that contributes to the control of attention during periods of distraction [1], [2]. In line with this view, pMFC activity is typically greater in incongruent than in congruent trials of response-interference (e.g., Stroop) tasks [3], [4], [5]. However, the nature of the process underlying this congruency effect remains unclear. We therefore sought to distinguish between two accounts of the congruency effect: the conflict monitoring account [6] and the time on task account [7].

Both accounts posit the congruency effect indexes a process whose recruitment increases with reaction time (RT) in response-interference tasks. The conflict monitoring account posits the effect is driven by conflict between competing response alternatives, which “more closely tracks reaction time (RT) than stimulus congruence condition when the two are dissociated ([8], p. 3).” The time on task account posits the effect is driven by a process whose recruitment increases with time on task independent of response conflict (e.g., sustained attention, arousal, effort, etc.) [7], [9], [10]. In line with both accounts, pMFC activity *within* the congruent and incongruent conditions increases linearly with RT on a trial-by-trial basis; consequently, the congruency effect vanishes after controlling for the within-condition relationship between pMFC activity and RT in response-interference tasks [7], [9].

To distinguish between the conflict monitoring and time on task accounts, we used functional MRI to record brain activity in twenty-four healthy adults while they performed two tasks: a response-interference task and a simple RT task with only one possible response. Demands on a process that detects response conflict should increase with RT in the response-interference task [8] but not in the simple RT task. Indeed, a simple RT task with only one possible response involves no response selection [11] and therefore cannot engender response conflict [7], [12]. In contrast, demands on a process whose recruitment increases with time on task independent of response conflict should increase with RT in both tasks.

Given these considerations, whether the congruency effect is more consistent with the conflict monitoring account or with the time on task account should become apparent after answering two questions. First, does pMFC activity increase with RT in the simple RT task the way it increases with RT in response-interference tasks [7], [9]? Second, if the first question is answered affirmatively, then does the congruency effect in the response-interference task vanish after controlling for the relationship between pMFC activity and RT in the simple RT task? The answers to both questions were yes. Thus, the congruency effect appears more consistent with the time on task account than with the conflict monitoring account.

## Materials and Methods

### Ethics Statement

All experimental procedures were approved by the University of Michigan’s Biomedical and Health Sciences Institutional Review Board and were in compliance with the Code of Ethics of the World Medical Association (Declaration of Helsinki). These procedures were fully described to each participant before he or she consented to take part in the study. Written informed consent was obtained from each participant.

### Participants

Twenty-six healthy adults from the University of Michigan community participated in the experiment. All were right-handed with normal or corrected-to-normal vision and reported no history of neurological or psychiatric disorder. Ultimately, two participants were excluded: one for excessive head motion and another for poor behavioral performance. Thus, the final sample included twenty-four participants (mean age: 21.0 years; range: 18 to 25 years; nine female).

### Tasks and Procedure

Participants performed a response-interference task - the multi-source interference task (MSIT) [13] - and a simple RT task in separate scanning runs. The order of the two tasks was counterbalanced across participants: half completed the MSIT first and the simple RT task second while the other half completed these tasks in the opposite order. There were four runs of the MSIT and two runs of the simple RT task. Each run contained 96 trials. In each trial, a stimulus was displayed for 500 ms. Participants were instructed to respond as quickly and as accurately as possible. Trials were separated by a stimulus onset asynchrony ranging from 2500 ms to 6250 ms. SOAs were chosen following a pseudo-exponential distribution [14].

**Figure 1:**
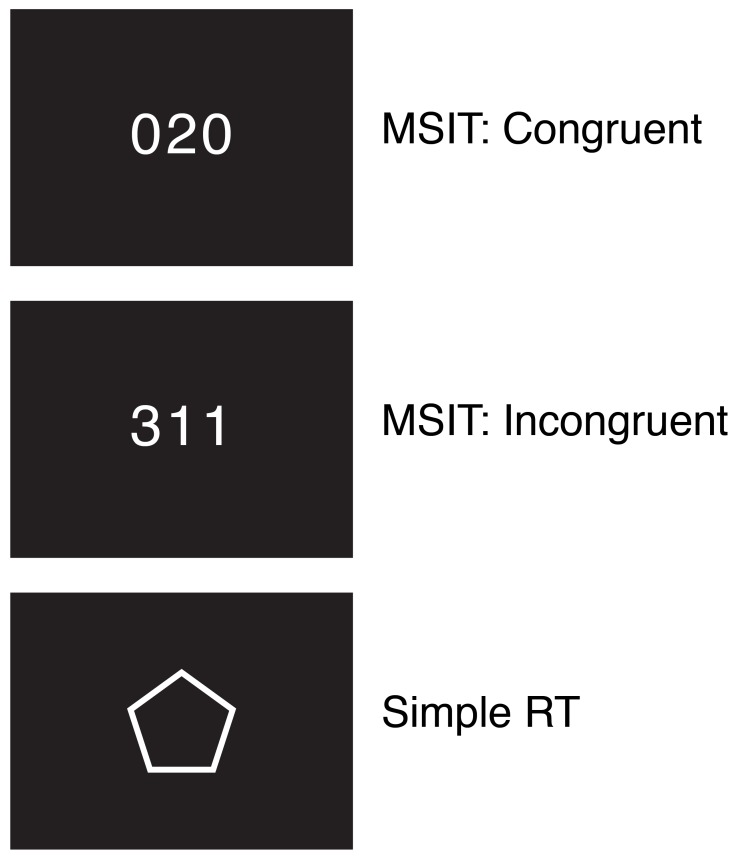
Example stimuli from the MSIT and the simple RT task. In each congruent or incongruent trial of the MSIT, participants indicated the identity of the unique digit (1, 2, or 3). In the simple RT task, they responded each time a pentagon appeared. In both tasks, participants were asked to respond as quickly and as accurately as possible each time a stimulus was presented (duration, 500 ms). Trials were separated by a stimulus onset asynchrony (SOA) that ranged from 2500 ms to 6250 ms following a pseudo-exponential distribution that favored short SOAs.

In each trial of the MSIT, participants viewed a horizontally- aligned row of three digits at the center of the screen: a unique target digit and two distractor digits. The distractor digits were identical to each other but different from the target digit. Participants were instructed to press the right thumb, right index finger, or right middle finger button on an MR-compatible response box, respectively, to indicate the target digit was a 1, 2, or 3. In congruent trials (**Fig. 1**, top), the distractor digits were 0 s, which did not map onto any of the response options. In addition, the position of the target digit was spatially compatible with its value (“100”, “020”, or “003”). In incongruent trials (**Fig. 1**, middle), the distractor digits were 1 s, 2 s, or 3 s, which mapped onto a response that competed with the correct response. Further, the position of the target digit was spatially incompatible with its value (e.g., “211”, “331”, or “232”).

In each trial of the simple RT task (**Fig. 1**, bottom), participants responded to a regular pentagon at the center of the screen. Responses were made with the right thumb, right index finger, or right middle finger (counterbalanced across participants).

### Data Acquisition

Neuroimaging data were collected using a GE Signa 3 T MRI scanner. Functional images were acquired using a reverse spiral sequence (repetition time: 1250 ms, echo time: 30 ms, flip angle: 90°, field of view: 220 by 220 mm, voxel dimensions: 3.44 by 3.44 by 4.5 mm; no slice gap). Each functional volume included 27 slices oriented parallel to the anterior commissure-posterior commissure plane. 297 functional volumes were collected in each run. To allow for stabilization of the BOLD signal, the first five volumes of each run were discarded prior to analysis (no trials were presented during these volumes). We monitored breath and heart rate during functional imaging for subsequent artifact reduction. Finally, we collected high-resolution T1-weighted spoiled gradient recalled (SPGR) images (repetition time: 10.5 ms; echo time: 3.4 ms; flip angle: 25°; field of view: 240 by 240 mm; voxel dimensions: 1.02 by 1.02 by 1.2 mm; 124 slices) and additional T1-weighted images in the same plane as the functional images (voxel dimensions: 0.86 by 0.86 by 4.5 mm; 27 slices) for subsequent spatial normalization.

### Data Preprocessing

Data preprocessing for each participant consisted of several steps. To begin, the functional images were corrected for fluctuations in breath and heart rate [15]. Next, the functional images were pre-processed using standard procedures and parameters in SPM8 (Wellcome Department of Cognitive Neurology, London, UK, www.fil.ion.ucl.ac.uk). First, each functional image was corrected for asynchronous timing of slice acquisition via a sinc interpolation that temporally aligned the BOLD signal in each slice to the BOLD signal in the first slice. Second, each functional image was motion-corrected by applying a rigid-body transformation that aligned the image with the first functional image that was collected. Third, each functional image was normalized to Montreal Neurological Institute (MNI) space using parameters generated by normalizing the high-resolution SPGR anatomical image. Normalizing the SPGR image involved coregistering it to the mean functional image using a two-stage procedure: in the first stage, the in-plane structural image was coregistered to the mean functional image; in the second stage, the SPGR image was coregistered to the in-plane structural image. Following coregistration, the SPGR image was skull-stripped using the VBM8 toolbox (http://dbm.neuro.uni-jena.de/vbm) and normalized to the skull-stripped MNI template provided by FSL (“MNI152_T1_1 mm_brain.nii”). Finally, each functional image was normalized to MNI space using the parameters generated by normalizing the SPGR image. Fourth, and last, each functional image was smoothed using a Gaussian kernel (8 mm full width at half maximum).

### Model Estimation

The preprocessed functional images were analyzed using the general linear model as implemented in SPM8. First, we included three regressors to model the average BOLD responses to correct congruent trials, correct incongruent trials, and correct simple RT trials. Each of these regressors was formed by convolving the onset times of the trials in the associated condition (e.g., congruent) with a canonical hemodynamic response function whose duration was zero seconds. Second, we included three linear, parametric RT regressors to model the trial-by-trial relationship between RT and the BOLD response within each of these conditions. Each RT regressor was mean-centered, rendering it orthogonal to its associated condition regressor, and convolved with a canonical hemodynamic response function whose duration was zero seconds. Errors, outliers, misses, and response omissions were modeled separately and excluded from further analyses (errors in the simple RT task were trials in which the participant failed to respond). Outliers were defined as trials with RTs more than three standard deviations away from their conditional mean. To account for the residual effects of head movement on the BOLD signal, we also included twenty-four head motion regressors as nuisance covariates. These were the linear, squared, time-shifted, and squared time-shifted transformations of the six translation and rotation parameters estimated during motion realignment. Finally, during model estimation, the data were temporally filtered using a 128 s high-pass cutoff and corrected for temporal autocorrelation using AR(1) modeling.

### RT-regression

As in our prior studies [9], [16], we used a within-participant RT-regression analysis to control for the trial-by-trial relationship between RT and the BOLD signal when contrasting activity in incongruent and congruent trials. First, using the model described earlier (see *Model Estimation*), we obtained an estimate of the degree to which the BOLD signal increased linearly with RT in congruent trials (i.e., from the parametric RT regressor for congruent trials). A separate estimate, or RT-BOLD slope (β_RT-Congruent_), was obtained for each voxel in the brain volume. Second, and most important, we used the RT-BOLD slope to estimate activity in congruent trials with RTs equal to the mean RT in incongruent trials (CongruentEQ_Congruent_ trials), separately for each voxel. Specifically, we multiplied each voxel’s RT-BOLD slope, β_RT-Congruent_, by the difference in mean RT between incongruent and congruent trials (Incongruent RT – Congruent RT) and added the resulting quantity to the regression-derived estimate of mean activity in correct congruent trials (Congruent). Thus, at each voxel, activity in CongruentEQ_Congruent_ (i.e., RT-equated congruent) trials was estimated as follows:
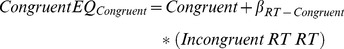
(1)


Finally, we contrasted activity in incongruent trials to that in CongruentEQ_Congruent_ trials.

In an analogous analysis, we controlled for the trial-by-trial relationship between RT and the BOLD signal when contrasting activity in incongruent and congruent trials using the RT-BOLD slope from simple RT trials (β_RT-Simple_). In this analysis, we estimated activity in RT-equated congruent trials as follows:
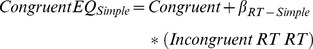
(2)


We then contrasted activity in incongruent trials to that in CongruentEQ_Simple_ trials.

In the RT-regression analyses above, we chose to model linear relationships between RT and the BOLD signal but not higher-order relationships. This choice maximized statistical power by minimizing the number of regressors in the design matrix. Moreover, this choice was justified by the dearth of higher-order (i.e., quadratic, cubic, and quartic) RT-BOLD relationships in our prior studies of response-interference tasks [9], [17], [18], [19].

### Group Analyses

Group analyses were conducted using random effects models implemented in SPM8. Whole-brain analyses used an FDR-corrected height threshold of *q* < = 0.05 and an extent threshold of *k* > = 20 voxels. Conjunction analyses revealed voxels that were activated in each of two whole-brain analyses. Region of interest (ROI) analyses compared parameter estimates for different conditions after averaging those estimates across all voxels within a sphere (radius, 6 mm). Orthogonal ROI analyses involved a sphere that was centered on coordinates identified in a previous study [20]. Non-orthogonal ROI analyses involved a sphere (radius, 6 mm) centered on a pMFC region that exhibited a highly significant congruency effect in the present study. T-tests conducted at the ROI level were considered significant if their associated p-values were less than 0.05 (two-tailed). Brain activations were overlaid on the Caret anatomical template [21] (http://brainmap.wustl.edu/caret). Code for the task presentation and data analysis is available at https://github.com/jmcarp/msit-rxn.

## Results

### Behavior

Replicating previous studies of the MSIT [9], [13], mean RT was slower in incongruent than in congruent trials (858 ms vs. 661 ms; *t*(23) = 13.18, *p*<0.001). Analogously, mean accuracy was lower in incongruent than in congruent trials (98.1% vs. 99.8%; *t*(23) = 5.21, *p*<0.001).

Also as expected, mean RT in the simple RT task was faster than mean RT in both incongruent trials (376 ms vs. 858 ms; *t*(23) = 18.45, *p*<0.001) and congruent trials (376 ms vs. 661 ms; *t*(23) = 17.62, *p*<0.001) of the MSIT. Mean accuracy in the simple RT task did not differ from mean accuracy in either congruent trials (98.9% vs. 99.8%; *t*(23) = −2.05, *p* = 0.051) or incongruent trials (98.9% vs. 98.1%; *t*(23) = 1.53, *p* = 0.14). We speculate that participants exhibited numerically higher error rates in the simple RT task than in congruent trials of the MSIT because the simple RT task was less engaging than the MSIT, leading participants to miss a target from time to time.

### FMRI

#### Whole-brain analyses of congruency effects and RT-BOLD relationships

**Figure 2:**
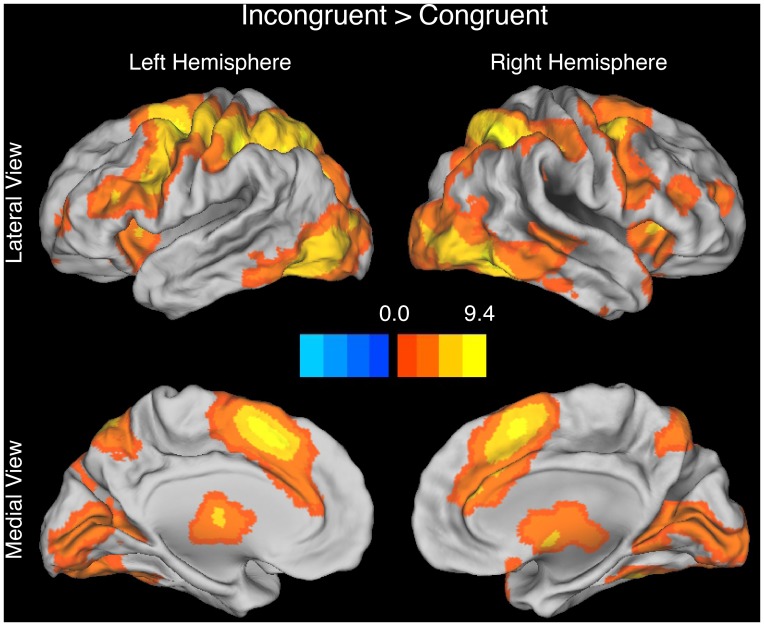
Brain activations revealed by the Incongruent>Congruent contrast in the MSIT. Consistent with both the time on task account and the conflict monitoring account, we observed prominent positive activations (i.e., congruency effects) in the posterior medial prefrontal cortex (pMFC) (medial brain views). The contrast map is rendered using an FDR-corrected height threshold of *q* < = 0.05 and an extent threshold of k > = 20 contiguous voxels. The orange-to-yellow scale indicates increasingly positive t-values while the dark-blue-to-light-blue scale indicates increasingly negative t-values. Activations are overlaid on the Caret anatomical template.

**Figure 3:**
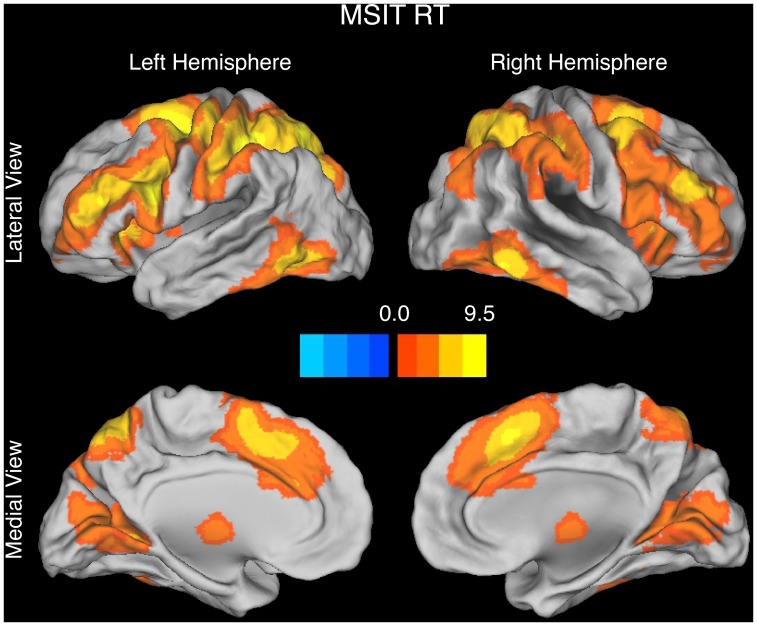
Brain activations revealed by the parametric contrast of RT in the MSIT. In line with both the time on task account and the conflict monitoring account, the BOLD signal varied linearly and positively with RT in the pMFC (medial brain views). The contrast map is rendered using an FDR-corrected height threshold of *q* < = 0.05 and an extent threshold of k > = 20 contiguous voxels. The orange-to-yellow scale indicates increasingly positive t-values while the dark-blue-to-light-blue scale indicates increasingly negative t-values. Activations are overlaid on the Caret anatomical template.

**Figure 4:**
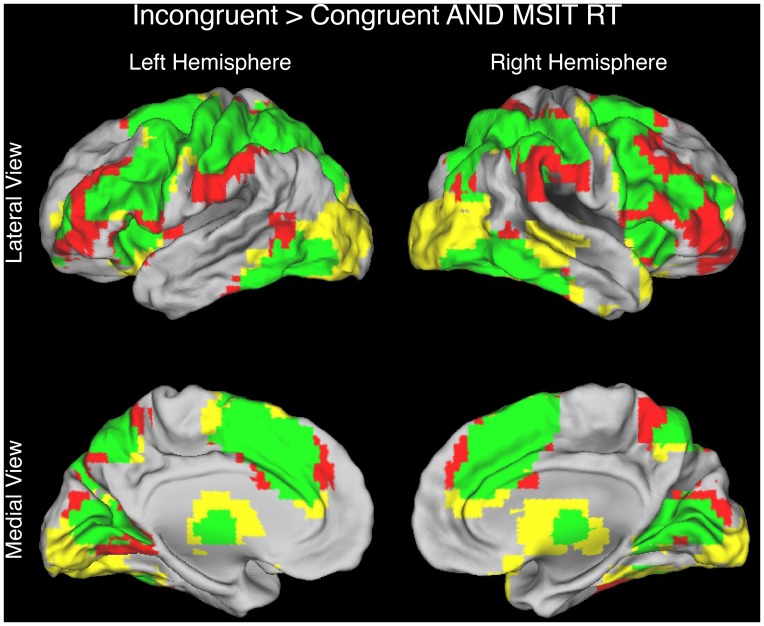
Conjunction analysis involving the Incongruent>Congruent contrast and the parametric contrast of RT in the MSIT. Consistent with the time on task account and with the conflict monitoring account, the pMFC was activated by both contrasts (medial brain views). Regions showing significant activation for only the Incongruent>Congruent contrast are highlighted in yellow (FDR-corrected height threshold, *q* < = 0.05; extent threshold, k > = 20 contiguous voxels), regions showing significant activation for only the parametric contrast of RT are highlighted in red (FDR-corrected height threshold, p = <0.05; extent threshold, k > = 20 contiguous voxels), and regions showing significant activation for both contrasts are highlighted in green.

Replicating our prior work [9], three findings in the MSIT were consistent with both the conflict monitoring account and the time on task account. First, incongruent trials evoked greater activity than congruent trials in frontal and parietal regions including the pMFC (**Fig.** **2**, **Table 1**). Second, averaging across the RT-BOLD slopes for congruent and incongruent trials, the BOLD signal varied linearly and positively with RT in frontal and parietal regions including the pMFC (**Fig.** **3** **,** **Table 2**). Third, a conjunction analysis revealed congruency effects and RT-BOLD relationships in overlapping frontal and parietal regions including the pMFC (**Fig.** **4**, **Table 3**).

**Table 1:** Brain activations revealed by the Incongruent>Congruent contrast in the MSIT.

Region	Number of voxels	MNI Coordinates			Peak *t*-value
		X	Y	Z	
Medial Frontal Gyrus	11896	−5.81	11.75	49.00	9.39
Left Middle Frontal Gyrus	11896	−29.88	−5.44	53.50	10.29
Right Middle Frontal Gyrus	11896	28.56	−2.00	58.00	7.63
Left Inferior Frontal Gyrus	11896	−29.88	25.50	−0.50	5.14
Right Inferior Frontal Gyrus	11896	32.00	18.62	4.00	5.17
Left Thalamus	11896	−9.25	−12.31	13.00	6.31
Right Thalamus	11896	11.38	−12.31	13.00	4.61
Left Superior Parietal Lobe	11896	−19.56	−60.44	44.50	11.70
Right Superior Parietal Lobe	11896	28.56	−57.00	49.00	9.53
Left Fusiform Gyrus	11896	−36.75	−63.88	−9.50	7.57
Right Inferior Temporal Gyrus	11896	52.62	−77.62	−5.00	8.05

**Table 2:** Brain activations revealed by the parametric contrast of RT in the MSIT.

Region	Number of voxels	MNI Coordinates			Peak *t*-value
		X	Y	Z	
Medial Frontal Gyrus	7108	4.50	15.19	49.00	8.12
Left Middle Frontal Gyrus	7108	−26.44	−5.44	53.50	8.26
Right Middle Frontal Gyrus	7108	25.12	4.88	53.50	10.18
Left Precentral Gyrus	7108	−43.62	4.88	22.00	8.49
Right Inferior Frontal Gyrus	7108	52.62	8.31	35.50	5.69
Left Thalamus	86	−12.69	−15.75	4.00	3.99
Right Thalamus	86	4.50	−15.75	−0.50	3.86
Left Inferior Parietal Lobule	7108	−36.75	−43.25	44.50	7.25
Right Inferior Parietal Lobule	7108	35.44	−43.25	58.00	5.12
Left Middle Occipital Gyrus	339	−50.50	−67.31	−9.50	6.35
Right Middle Temporal Gyrus	398	56.06	−53.56	−14.00	7.47
Left Lingual Gyrus	413	−19.56	−53.56	−0.50	4.34
Right Lingual Gyrus	413	18.25	−50.12	−0.50	3.30

**Table 3:** Conjunction analysis involving the Incongruent>Congruent contrast and the parametric contrast of RT in the MSIT.

Region	Number of voxels	MNI Coordinates			Peak *t*-value
		X	Y	Z	
Medial Frontal Gyrus	3744	4.50	11.75	49.00	7.66
Left Middle Frontal Gyrus	3744	−26.44	−5.44	53.50	8.26
Right Middle Frontal Gyrus	3744	28.56	1.44	49.00	6.98
Left Inferior Frontal Gyrus	3744	−33.31	22.06	−0.50	5.14
Right Inferior Frontal Gyrus	3744	32.00	18.62	4.00	5.19
Left Thalamus	86	−12.69	−15.75	4.00	3.99
Right Thalamus	86	7.94	−15.75	−0.50	3.67
Left Superior Parietal Lobule	3744	−19.56	−67.31	44.50	8.08
Right Superior Parietal Lobule	827	25.12	−63.88	49.00	6.47
Left Middle Occipital Gyrus	229	−50.50	−67.31	−9.50	6.24
Left Inferior Temporal Lobe	298	52.62	−57.00	−14.00	6.01
Left Lingual Gyrus	296	−19.56	−57.00	−0.50	3.90
Right Lingual Gyrus	296	18.25	−50.12	−0.50	3.30

**Figure 5:**
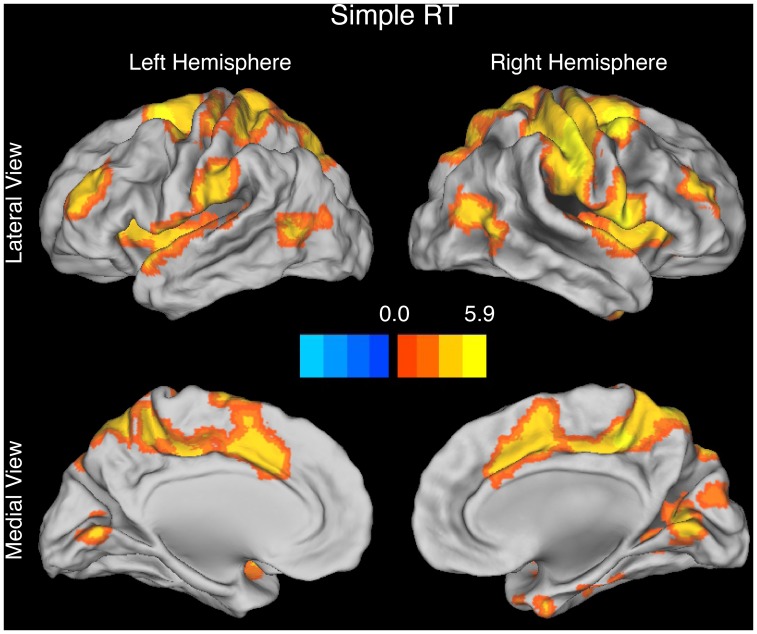
Brain activations revealed by the parametric contrast of RT in the simple RT task. More consistent with the time on task account than with the conflict monitoring account, the BOLD signal varied linearly and positively with RT in the pMFC (medial brain views). The contrast map is rendered using an FDR-corrected height threshold of *q* < = 0.05 and an extent threshold of k > = 20 contiguous voxels. The orange-to-yellow scale indicates increasingly positive t-values while the dark-blue-to-light-blue scale indicates increasingly negative t-values. Activations are overlaid on the Caret anatomical template.

**Figure 6:**
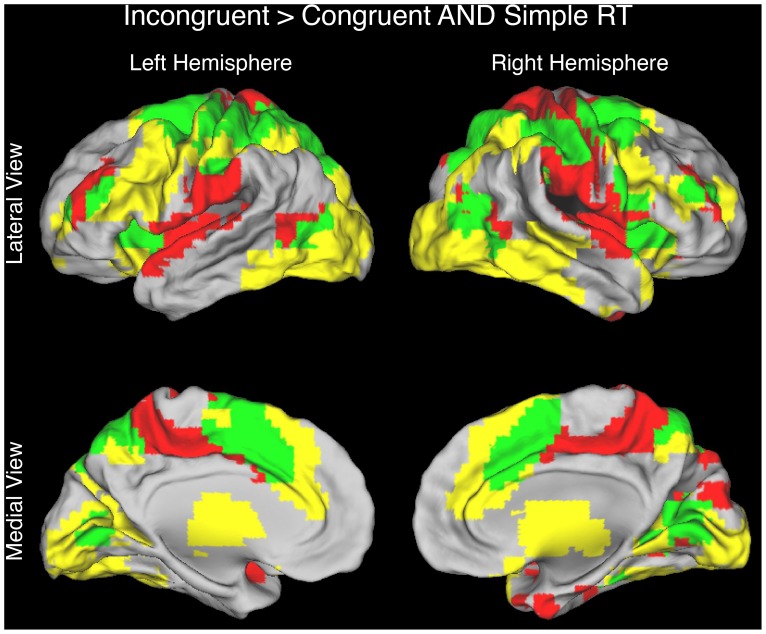
Conjunction analysis involving the Incongruent>Congruent contrast in the MSIT and the parametric contrast of RT in the simple RT task. More consistent with the time on task account than with the conflict monitoring account, the pMFC was activated by both contrasts (medial brain views). Regions showing significant activation for only the Incongruent>Congruent contrast are highlighted in yellow (height threshold, *q* < = 0.05; extent threshold, k > = 20 contiguous voxels), regions showing significant activation for only the parametric contrast of RT are highlighted in red (height threshold, *q* < = 0.05; extent threshold, k > = 20 contiguous voxels), and regions showing significant activation for both contrasts are highlighted in green.

Other findings, however, appeared more consistent with the time on task account than with the conflict monitoring account. First, in the simple RT task, the BOLD signal varied linearly and positively with RT in frontal and parietal regions including the pMFC (**Fig. 5**, **Table 4**). Second, a conjunction analysis revealed that many of these regions – including the pMFC – also exhibited a congruency effect in the MSIT (**Fig. 6**, **Table 5**), suggesting that a process whose recruitment increases with time on task contributes to this effect [7]. Third, a whole-brain analysis contrasting the RT-BOLD slope in the MSIT (averaging across the RT-BOLD slopes for congruent and incongruent trials) to that in the simple RT task revealed no slope differences, suggesting that a process whose recruitment increases with time on task might fully account for the congruency effect.

**Table 4:** Brain activations revealed by the parametric contrast of RT in the simple RT task.

Region	Number of voxels	MNI Coordinates			Peak *t*-value
		X	Y	Z	
Cingulate Gyrus	4544	−9.25	8.31	40.00	4.13
Left Superior Frontal Lobe	4544	−19.56	−2.00	53.50	6.02
Right Superior Frontal Lobe	4544	18.25	1.44	58.00	5.27
Left Inferior Frontal Gyrus	4544	−33.31	15.19	4.00	3.95
Right Inferior Frontal Gyrus	4544	35.44	11.75	4.00	4.33
Left Middle Frontal Gyrus	4544	−33.31	35.81	22.00	3.99
Right Middle Frontal Gyrus	4544	32.00	32.38	22.00	4.33
Left Postcentral Gyrus	4544	−33.31	−43.25	62.50	4.48
Right Postcentral Gyrus	4544	25.12	−50.12	67.00	3.92
Left Middle Temporal Gyrus	78	−47.06	−60.44	4.00	4.25
Right Middle Temporal Gyrus	128	42.31	−63.88	13.00	4.10
Cuneus	127	4.50	−70.75	4.00	4.14

**Table 5:** Conjunction analysis involving the Incongruent>Congruent contrast in the MSIT and the parametric contrast of RT in the simple RT task.

Region	Number of voxels	MNI Coordinates			Peak *t*-value
		X	Y	Z	
Superior Frontal Gyrus	1339	1.06	4.88	53.50	3.53
Left Superior Frontal Gyrus	1339	−19.56	−2.00	53.50	6.01
Right Middle Frontal Gyrus	1339	28.56	1.44	49.00	6.47
Left Inferior Frontal Gyrus	103	−33.31	15.19	8.50	3.95
Right Inferior Frontal Gyrus	224	35.44	15.19	4.00	4.31
Left Postcentral Gyrus	1339	−40.19	−32.94	58.00	4.73
Right Postcentral Gyrus	203	45.75	−29.50	44.50	5.19
Left Precuneus	1339	−16.12	−63.88	49.00	4.27
Right Precuneus	100	11.38	−63.88	58.00	4.22
Right Middle Temporal Gyrus	58	52.62	−67.31	−0.50	3.40
Cuneus	106	4.50	−70.75	4.00	4.18

#### Whole-brain analyses of congruency effects after controlling for RT-BOLD relationships

Consistent with the time on task account, the foregoing analyses revealed that pMFC activity increased with RT in the simple RT task just as it did in the MSIT. Thus, we next investigated whether pMFC activity increased with RT in the simple RT task enough to fully account for the congruency effect in the MSIT. More generally, we investigated whether controlling for the RT-BOLD relationship in either the MSIT or the simple RT task eliminates the congruency effect in the pMFC. As reviewed in the Introduction, both the conflict monitoring account and the time on task account predict the congruency effect will be reduced after controlling for the RT-BOLD relationship in the MSIT. However, only the time on task account predicts such a reduction after controlling for the RT-BOLD relationship in the simple RT task.

First, we contrasted activity in incongruent and congruent trials after equating these conditions for RT using the RT-BOLD slope in congruent trials. That is, we compared mean activity in incongruent trials to activity in RT-equated, CongruentEQ_Congruent_ trials. This RT-corrected contrast yielded no significant activations in the brain. Thus, in line with both accounts, the congruency effect in the pMFC vanished after controlling for the RT-BOLD relationship in congruent trials.

Second, we contrasted activity in incongruent and congruent trials after equating these conditions for RT using the RT-BOLD slope in the simple RT task. That is, we compared mean activity in incongruent trials to activity in RT-equated, CongruentEQ_Simple_ trials. This RT-corrected contrast revealed no significant activations in the brain. Thus, in line with the time on task account but not with the conflict monitoring account, the congruency effect in the pMFC vanished after controlling for the RT-BOLD relationship in the simple RT task.

#### Orthogonal ROI analyses

The corrections for multiple comparisons in the whole-brain analyses may have pushed small RT-corrected congruency effects beneath the threshold of statistical significance. Thus, to increase statistical power, we conducted region of interest (ROI) analyses in three ROIs that a prior meta-analysis implicated in interference detection and control [20]: the pMFC (MNI coordinates: *x* = 2, *y = *16, *z* = 46), bilateral inferior frontal gyrus (IFG; MNI coordinates: left IFG: *x* = −36, *y* = 16, *z* = 4; right IFG: *x* = 44, *y* = 14, *z* = 8), and bilateral inferior parietal lobule (IPL; MNI coordinates: left IPL: *x* = −36, *y* = −56, *z* = 44; right IPL: *x* = 40, *y* = −52, *z* = 42). As in the whole-brain analyses, we evaluated the congruency effect in each ROI (a) before controlling for any RT-BOLD relationships, (b) after controlling for the linear RT-BOLD relationship in congruent trials, and (c) after controlling for the linear RT-BOLD relationship in simple RT trials.

The results of the ROI analyses are presented in Table 6. In all five ROIs, there was a highly significant congruency effect in the original data (all *p’s* <0.005, two-tailed). Moreover, in line with the whole-brain results, incongruent trials did not evoke greater activity than congruent trials after controlling for the RT-BOLD relationship in either (a) congruent trials or (b) simple RT trials (in fact, the opposite result was observed in bilateral IFG after controlling for the RT-BOLD relationship in simple RT trials).

**Table 6:** Region of interest analyses of the Incongruent>Congruent contrast in (a) the original data, (b) after correcting for the RT-BOLD relationship in congruent trials of the MSIT, and (c) after correcting for the RT-BOLD relationship in simple RT trials.

Region of interest	Incongruent>Congruent		
	Original data	After correcting for the RT-BOLD relationship in congruent trials	After correcting for the RT-BOLD relationship in simple RT trials
Posterior Medial Frontal Cortex	*t* = 7.68****	*t* = 1.87	*t* = − 0.71
Left Inferior Frontal Gyrus	*t* = 5.01****	*t* = 0.01	*t* = − 2.36*
Right Inferior Frontal Gyrus	*t* = 3.22***	*t* = 0.53	*t* = − 2.35*
Left Inferior Parietal Lobule	*t* = 6.25****	*t* = 0.16	*t* = 1.85
Right Inferior Parietal Lobule	*t* = 4.54***	*t* = − 0.57	*t* = 0.42

* p<0.05.

** p<0.01.

*** p<0.005.

**** p<0.0001.

**Figure 7:**
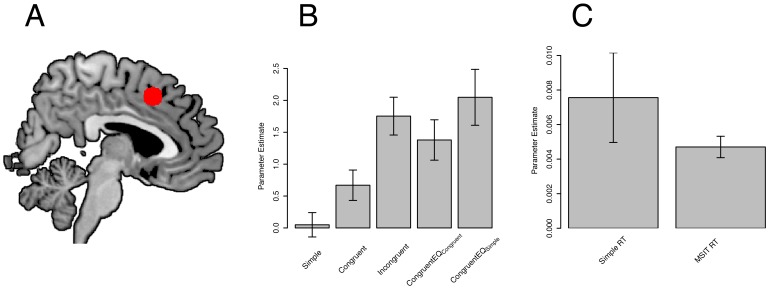
Activations revealed by region of interest analyses in the posterior medial frontal cortex (pMFC). Panel A: The pMFC ROI overlaid on the ch2better anatomical template. Panel B: PMFC activation in the Simple, Congruent, Incongruent, CongruentEQ_Congruent_, and CongruentEQ_Simple_ conditions. Consistent with the time on task account but not with the conflict monitoring account, the congruency effect in the MSIT was completely eliminated after controlling for the within-condition relationship between pMFC activity and RT in the simple RT task. Indeed, as shown in the Figure, Incongruent trials evoked numerically *less* activity than CongruentEQ_Simple_ trials. Panel C: Parametric contrasts of RT in the simple RT task and the MSIT (averaged across the congruent and incongruent conditions). Consistent with the time on task account but not with the conflict monitoring account, the BOLD signal in the pMFC varied linearly and positively with RT in both tasks. Furthermore, the magnitude of this RT-BOLD relationship did not differ significantly between the two tasks. Error bars indicate the standard error of the mean.

Since the most important region for present purposes is the pMFC, we now discuss a few additional aspects of the data from this ROI (**Fig. 7a** **)**. First, the congruency effect was completely eliminated (and even non-significantly reversed) after controlling for the RT-BOLD relationship in simple RT trials (**Fig. 7b**; compare the middle bar to the far right bar). Second, we observed a significant positive RT-BOLD relationship not only in the MSIT (*t*(23) = 7.58, *p*<0.0001) but also in the simple RT task (*t*(23) = 2.92, *p*<0.01) (**Fig. 7c** **)**, and these RT-BOLD relationships did not differ (*t*(23) = −1.08, *ns*). These findings provide further evidence that the congruency effect in the pMFC is more consistent with the time on task account than with the conflict monitoring account.

#### Non-orthogonal ROI analyses

One might wonder whether similar effects were present even in the pMFC region that showed the most significant congruency effect in the whole-brain analysis. To answer this question, we created a new ROI centered on this coordinate (*x* = − 5.81, *y* = 11.75, *z* = 49; **Table 1**). As this coordinate was chosen precisely because it showed a highly significant congruency effect in the whole-brain analysis, subsequent ROI analyses were biased to yield a significant congruency effect after controlling for the RT-BOLD relationship in either (a) the MSIT or (b) the simple RT task. For this reason, the presence of a congruency effect after controlling for either of these RT-BOLD relationships would not be particularly informative. However, an elimination of the congruency effect after controlling for the RT-BOLD relationship in the simple RT task would provide especially strong support for the time on task account.

The results of the non-orthogonal ROI analyses were as follows. First, confirming the whole-brain analysis, there was a significant congruency effect (*t*(23) = 9.13, *p*<0.0001). Second, although the congruency effect remained significant after controlling for the RT-BOLD relationship in the MSIT (*t*(23) = 2.53, *p*<0.02), it was completely eliminated (and even non-significantly reversed) after controlling for the RT-BOLD relationship in the simple RT task (*t*(23) = − 0.38, *ns*). Third, we observed a significant positive RT-BOLD relationship not only in the MSIT (*t*(23) = 5.52, *p*<0.0001) but also in the simple RT task (*t*(23) = 3.09, *p*<0.01), and these RT-BOLD relationships did not differ (*t*(23) = −1.31, *ns*). These findings further indicate that the congruency effect in the pMFC appears more consistent with the time on task account than with the conflict monitoring account.

## Discussion

We sought to distinguish between two accounts of the congruency effect that is ubiquitously observed in the pMFC during response-interference (e.g., Stroop) tasks. The conflict monitoring account posits the effect is driven by a process that detects response conflict, which is indexed by trial-specific RT in response-interference tasks [8]. The time on task account posits the effect is driven by a process whose recruitment increases with time on task independent of response conflict (e.g., sustained attention, arousal, effort, etc.) [7], [9], [10]. As described next, our findings weigh in favor of the time on task account.

### The Congruency Effect in the pMFC Appears More Consistent with the Time on Task Account than with the Conflict Monitoring Account

To distinguish between the conflict monitoring and time on task accounts, we contrasted the RT-BOLD relationship in a response-interference task to that in a simple RT task with only one possible response. Demands on a process that detects response conflict should increase with RT in a response-interference task [8] but not in a simple RT task [7], [12]. Indeed, a simple RT task involves no response selection [11] and therefore cannot engender response conflict [7], [12]. On the other hand, demands on a process whose recruitment increases with time on task independent of response conflict should increase with RT in both tasks [7], [9], [10]. Given these considerations, we reasoned that if the RT-BOLD relationship in the pMFC is driven by response conflict [8], then it should be present in a response-interference task but not in a simple RT task. Alternatively, if it is driven by time on task [7], then it should be present in both tasks.

Our findings appeared more consistent with the time on task account of the congruency effect than with the conflict monitoring account. First, the RT-BOLD relationship in the pMFC was as robust in the simple RT task as in the MSIT. Second, the congruency effect in the pMFC vanished after controlling for the RT-BOLD relationship in the simple RT task. These findings appear to provide novel support for the time on task account.

One might argue, however, that the simple RT task could have engendered as much response conflict as the MSIT. In line with this possibility, the conflict monitoring account has been extended to certain tasks that involve only one possible response [6], [22], [23], [24]. These tasks require participants to respond to the majority of stimuli while inhibiting a response to a few (the go/no-go task) or to respond to a few stimuli while inhibiting a response to the majority (the reverse go/no-go task). Notably, low-frequency responses in these tasks are thought to engender response conflict because they involve overcoming a pre-potent tendency to perform the opposing action. Such conflict could not have been present in our simple RT task, however, because each stimulus required the same “go” response.

One might therefore argue that some other form of conflict could have produced the RT-BOLD relationship in the simple RT task. However, this argument renders the concept of conflict nearly impossible to falsify (and of questionable utility) because it attributes any increase in RT to an unspecified conflict between mental representations. Thus, in the absence of strong evidence showing that our simple RT task engenders response conflict, the present findings appear more consistent with the time on task account than with the conflict monitoring account.

Finally, one might argue that stronger evidence linking the pMFC to conflict detection comes from brain imaging methodologies that provide higher temporal resolution than functional MRI. However, much of the evidence from such methodologies is equivocal. First, while an influence of congruency sequence effects on pMFC activity in certain EEG and single-cell recording studies may appear to suggest a role for the pMFC in detecting response conflict [25], [26], these effects were confounded with stimulus and/or response repetitions that likely recruited other processes [27], [28], [29]. Second, although the Error Related Negativity (ERN) in EEG studies has been posited to index a process that detects response conflict (i.e., the co-activation of competing motor responses) [30], electromyographic (EMG) recordings have revealed that ERN amplitude *decreases* (rather than increases) with the temporal overlap of competing response activations [31]. Third, although greater theta power over the medial frontal cortex in error trials compared to RT-matched correct trials is consistent with a role for the pMFC in detecting response conflict [32], it may also index other processes that are recruited just after an error, such as processes that reorient attention [33], [34] or support affective responses [35]. In sum, while brain imaging methodologies featuring high temporal resolution may ultimately link the pMFC to a process that detects response conflict, much of the available evidence does not provide compelling support for this view.

### Implications for the Conflict Monitoring Account

The present findings suggest two potential limitations of the conflict monitoring account. First, as discussed earlier, the account may incorrectly assume that the congruency effect in the pMFC indexes a process that detects response conflict. In this scenario, one might posit that a different frontal or parietal region underlying control is responsible for detecting response conflict. While we cannot rule out this possibility, our finding that congruency effects were absent in all frontal and parietal regions after we controlled for the RT-BOLD relationship in the simple RT task provides no support for this view.

The second potential limitation of the conflict monitoring account is that it may incorrectly assume that within-condition RT variability in response-interference tasks indexes varying levels of response conflict. In the words of this account’s creators,

“…slow congruent trials are not slow *despite* having low conflict, and fast incongruent trials are not fast *despite* having high conflict. To the contrary, slow congruent trials are slow precisely *because* conflict is high – a consequence of failing to focus attention, misperceiving the distracter, or preparing the wrong response, etc. –while fast incongruent trials are fast *because* conflict is low ([8], pp. 3–4).”

This assumption appears difficult to reconcile with our finding that the RT-BOLD relationship in the pMFC was as robust in the simple RT task as is the MSIT. Thus, our findings suggest at least one of two core assumptions made by the conflict monitoring account is incorrect.

### The Present Study in Relation to Prior Studies of the pMFC

The present study complements a recent functional MRI study whose results also appeared incompatible with the conflict monitoring account of the congruency effect [7]. In this prior study, one group of participants performed a typical Stroop task. A second group performed a modified simple RT task, which involved responding each time a flashing checkerboard – whose duration varied across trials - disappeared. Analogous to the present findings, in the Stroop task there was a congruency effect in the pMFC. Further, in the simple RT task, pMFC activity increased with stimulus duration. However, two study limitations precluded a firm conclusion as to whether pMFC activity related to the stimulus duration effect (which presumably did not index response conflict) could fully account for the congruency effect in the Stroop task. First, the between-participants design was not optimal for determining whether these effects activated identical regions of the pMFC. Second, the congruency effect was never assessed after controlling for the effect of stimulus duration on pMFC activity. Given these limitations, the present study appears to provide stronger evidence that the time on task account provides a better explanation of the congruency effect than the conflict monitoring account.

The present study should also be interpreted in the context of prior work suggesting regional specialization in the pMFC. For instance, this work suggests regional specialization for processes that track error likelihood [36], voluntarily orient attention [37], and support the conscious experience of negative affect [38]. Given such specialization of function, our findings should not be viewed as indicating the entire pMFC is dedicated to a single process whose recruitment increases with time on task. Indeed, it is important to entertain the possibility that multiple RT-dependent processes contribute to the RT-BOLD relationships we have observed. For example, these relationships could reflect processes that support sustained attention [10], autonomic arousal [39], conscious effort [40], or various aspects of decision-making [41]. Future studies should investigate these and other possible contributors to RT-BOLD relationships.

### Broader Relevance of RT-BOLD Relationships

The prevalence of RT-BOLD relationships in frontal and parietal regions has led some researchers to question the interpretability of brain activity in these regions [7], [9], [10], [42]. Specifically, these researchers have argued that when conditional differences in brain activity are accompanied by conditional differences in mean RT, they may reflect either the process under investigation (e.g., response conflict) or a different process whose recruitment increases with time on task (e.g., sustained attention, arousal, or effort). In this context, our study is important because it illustrates how incorporating RT-BOLD relationships into an experimental design can help to clarify the nature of the process that is indexed by brain activity. Given the ubiquity of RT-BOLD relationships in frontal and parietal regions, future studies aimed at identifying the processes that drive these relationships may be crucial for furthering our understanding of how these regions contribute to cognitive processes.

### Conclusions

Our findings provide novel insight into the congruency effect that is typically observed in the pMFC during response-interference tasks. Specifically, they indicate that this effect appears less consistent with a process that detects response conflict than with a process whose recruitment increases with time on task independent of response conflict (e.g., sustained attention, arousal, effort, etc.). Future studies investigating the specific process or processes that drive RT-BOLD relationships in the pMFC may further advance our knowledge of how this brain region contributes to the control of attention.
